# Breast Density and Estradiol Are Major Determinants for Soluble TNF-TNF-R Proteins *in vivo* in Human Breast Tissue

**DOI:** 10.3389/fimmu.2022.850240

**Published:** 2022-03-30

**Authors:** Jimmy Ekstrand, Maja Zemmler, Annelie Abrahamsson, Peter Lundberg, Mikael Forsgren, Charlotta Dabrosin

**Affiliations:** ^1^ Department of Oncology, Linköping University, Linköping, Sweden; ^2^ Department of Biomedical and Clinical Sciences, Linköping University, Linköping, Sweden; ^3^ Department of Radiology, Linköping University, Linköping, Sweden; ^4^ Department of Medical and Health Sciences, Linköping University, Linköping, Sweden; ^5^ Center for Medical Image Science and Visualization (CMIV), Linköping University, Linköping, Sweden

**Keywords:** mammography, microdialysis, mammary gland, breast cancer, sex steroids, estradiol

## Abstract

High mammographic density and exposure to sex steroids are independent risk factors for breast cancer by yet unknown mechanisms. Inflammation is one hallmark of cancer and the tumor necrosis factor family of proteins (TNFSFs) and receptors (TNFRSFs) are key determinants of tissue inflammation. The relationship between TNFSFs/TNFRSFs and breast tissue density or local breast estradiol levels is unknown. We investigated whether TNFSFs and soluble TNFRSFs (sTNFRSFs) are dysregulated *in vivo* in human breast cancer and dense breast tissue of postmenopausal women. We explored TNFSF/TNFRSF correlations with breast density and estradiol, both locally in the breast and in abdominal subcutaneous (s.c.) fat as a measure of systemic effects. Microdialysis was used for local sampling of *in vivo* proteins and estradiol in a total of 73 women; 12 with breast cancer, 42 healthy postmenopausal women with different breast densities, and 19 healthy premenopausal women. Breast density was determined as lean tissue fraction (LTF) using magnetic resonance imaging. Microdialysis was also performed in estrogen receptor (ER) positive breast cancer in mice treated with the pure anti-estrogen fulvestrant and tumor tissue was subjected to immunohistochemistry. 23 members of the TNFSF/sTNFRSF families were quantified using proximity extension assay.Our data revealed upregulation of TNFSF10, 13 and 13B, TNFRSF6, 6B, 9, 11A, 11B, 13B, 14, and 19, and TNFR-1 and -2 in ER+ breast cancer in women. In dense breast tissue TNFSF10, 13, and 14, TNFRSF3, 6, 9, 10B, 13B, 14, 19, and TNFR-1 and -2 were upregulated. Certain TNFSFs/TNFRSFs were increased in premenopausal breasts relative to postmenopausal breasts. Furthermore, estradiol correlated with most of the TNFSF/sTNFRSF members, though LTF only correlated with some of the proteins. Several of these associations were breast tissue-specific, as very few correlated with estradiol in abdominal s.c. fat. Estrogen dependent regulations of TNFSF2 (TNF-α) and TNF-R2 were corroborated in ER+ breast cancer in mice. Taken together, our data indicate TNFSFs/sTNFRSFs may represent potential targetable pathways for treatment of breast cancer patients and in prevention of breast cancer development in women with dense breasts.

## Introduction

Dense breast tissue, as depicted on mammography, is a major independent risk factor for breast cancer; women with dense breasts have a 4-6-fold increased risk of the disease as compared to women with nondense breasts (1, 2). An inversed association of the absolute amount of nondense area and risk of breast cancer has also been shown (2). It has been suggested that 30% of all breast cancer cases occur in women with > 50% dense area (1, 3). Despite these associations, the biological mechanisms underlying the increased risk are poorly understood and no preventive therapy to these women is available. The major histological difference between normal breast tissue of varying density is higher amounts of stroma, including collagen, in dense breasts and higher amounts of fat in nondense breasts (4, 5). Approximately 5-10% of the tissue area in normal breasts comprise epithelial cells and there are no conclusive data on differences between dense and nondense breast regarding the quantity or proliferation rate of these cells (4–8). Thus, the microenvironment surrounding the epithelial cells is a key determinant in breast cancer initiation and progression. Exposure to sex steroids including estrogens is an established risk factor for breast cancer (9, 10). However, no association between circulating estrogen levels and breast density has been determined (1).

Adipose tissue is a major component in breast tissue and adipocytes, in concert with immune cells, are major contributors to tumor necrosis factor (TNF) production and secretion. TNF-α is a member of the TNF superfamily (TNFSF) with number 2 of this family, TNFSF2. TNFSFs binds to proteins in the TNF receptor superfamily (TNFRSF) (11). TNFSFs/TNFRSFs act as local regulators in a paracrine/autocrine fashion and the biological activity reflects the balance between these regulators in the microenvironment. Consequently, the local regulation of the TNF system is complex and its role in cancer is controversial, and both tumor-promoting and tumor-inhibiting actions have been detected. However, TNFSF2 has been shown to be a key player in tumor-stroma inflammation in breast cancer (12). As these proteins are released and cleaved locally in the tumor microenvironment into soluble proteins, mRNA analysis or immunohistochemistry cannot detect, quantify, or reveal the intricate and complex interplay that occurs in the extracellular tissue microenvironment. How TNFSFs/TNFRSFs are dysregulated in breast cancer or affected by tissue density in normal breasts is not fully understood and the role of estradiol in their regulation is today undetermined.

Here we provide novel data showing that the majority of TNFSF and sTNFRSF were significantly dysregulated in human breast cancer. In healthy postmenopausal women, normal dense breast tissue compared to normal nondense breasts, exhibited similar dysregulation as breast cancers. Additionally, premenopausal breast tissue displayed increased levels of several of these proteins as compared to postmenopausal breasts. Furthermore, we show that breast density, determined with a continuous measure density using on magnetic resonance imaging (MRI), correlated with some of the proteins, whereas estradiol correlated with the majority of the TNFSF/sTNFRSF family members. Additionally, several of these associations were breast tissue specific as only a minority of the proteins correlated with estradiol in abdominal subcutaneous fat (s.c.). Thus, estradiol seems to play a prominent role for the local control of TNFSF/sTNFRSF family members in normal human breast tissue *in vivo*. Our results identify the TNFSF/sTNFRSF family as potential targets for breast cancer therapeutics and for prevention measures to postmenopausal women with dense breasts and for estrogen dependent breast cancer progression.

## Materials and Methods

### Subjects

The study was carried out in accordance with the Declaration of Helsinki and the Regional Ethical Review Board of Linköping, Sweden approved the study. All subjects gave informed consent. A total of 73 women were included in the study. Of these 12 women with estrogen receptor positive breast cancer were investigated with microdialysis before surgery.

Forty-two healthy postmenopausal women (ages 55–74 years) were consecutively recruited for the study as previously described (13). In brief, on the mammography screening facility at Linköping University Hospital, mammograms of postmenopausal women were categorized according to the Breast Imaging Reporting and Data System (BI-RADS) (14). Women with either entirely fatty nondense (BI-RADS A) or extremely dense (BI-RADS D) were invited to the study. After informed consent, included women underwent magnetic resonance imaging (MRI) for calculations of lean tissue fraction (LTF) as a continuous measure of breast density, as previously described (13). LTF was calculated in a volume selection of 30 x 30 x 30 mm within the glandular tissue in the upper lateral quadrant of the left breast. After a second review of the mammograms two women had been miscategorized and were not dense or nondense. These two women were not included in the analyses.

Additionally, 19 nulliparous premenopausal women (ages 20–32 years) with a history of regular menstrual cycles (cycle length, 27–34 days) were included. None of the healthy volunteer women had a history of breast cancer or were currently using (or had used within the past 3 months) hormone replacement therapy, sex steroid-containing contraceptives, anti-estrogen therapies, including selective estrogen receptor modulators, or degraders.

### Microdialysis Procedure

Prior to insertion of the microdialysis catheters 0.5 mL lidocaine (10 mg*/*mL) was administrated intracutaneously. Microdialysis catheters (M Dialysis AB, Stockholm, Sweden), which consisted of a tubular dialysis membrane (diameter 0.52mm, 100,000 atomic mass cut-off) glued to the end of a double-lumen tube were inserted *via* a splitable introducer (M Dialysis AB), connected to a microinfusion pump (M Dialysis AB) and perfused with 154 mmol*/*L NaCl and 60g/L hydroxyethyl starch (Voluven^®^; Fresenius Kabi, Uppsala, Sweden), at 0.5 µL/min. The women with ongoing breast cancer were investigated with 10 mm long membranes; one catheter was inserted within the cancer tissue and the other into normal adjacent breast tissue. The healthy volunteer women were investigated with 20-mm long microdialysis membranes; one was placed in the upper lateral quadrant of the left breast and directed towards the nipple and the other in abdominal s.c. fat as previously described (15–25). The premenopausal women were subjected to microdialysis in the luteal phase of the menstrual cycle. The microdialysis catheter was placed in the same quadrant where LTF was determined.

After a 60-min equilibration period, the outgoing perfusate was stored at -80°C for subsequent analysis.

### Breast Cancer Model

The Institutional Animal Ethics Committee at Linköping University approved this study, which conformed to regulatory standards of animal care. Oophorectomized athymic mice (Balb/C-nu/nu, 6-8 weeks old, Scanbur, Sweden) were housed at Linköping University in ventilated cages with a light/dark cycle of 12/12 hours with rodent chow and water available *ad libitum*. Mice were anesthetized *via* intraperitoneal (i.p.) injection of ketamine/xylazine and implanted with a subcutaneous (s.c.) 3-mm pellet containing either 17β-estradiol (0.18 mg/60-day release, Innovative Research of America, Sarasota, FL, USA) or placebo. The active pellet releases serum concentrations of 150-250 pM estradiol (26). 5 × 10^6^ MCF-7 cells were injected into the dorsal mammary fat pads in 200 µl PBS. MCF-7 cells require estrogen for tumor formation and growth in mice, therefore, a non-estrogen control group is not possible to achieve. When tumors reached ≈20 mm^2^ in size the mice were treated with fulvestrant (5 mg/mouse twice per week, s.c.) in addition to the estradiol exposure.

### Microdialysis in Mice

Tumor-bearing mice with size-matched tumors were anesthetized with i.p. injections of ketamine/xylazine and maintained by repeated s.c. injections of ketamine/xylazine. Body temperatures were maintained using a heat lamp. Microdialysis probes with 4-mm membranes (CMA 20, 100-kDa cutoff; CMA Microdialysis AB, Kista, Sweden) were inserted into tumor tissue and connected to a microdialysis pump (CMA 102; CMA Microdialysis AB) perfused at 0.6 μl/min with 154 mmol*/*L NaCl and 60g/L hydroxyethyl starch (Voluven^®^; Fresenius Kabi, Uppsala, Sweden), as previously described (27, 28). After a 60-minute equilibrium period, outgoing perfusates (i.e., microdialysates) were collected and stored at -80°C for subsequent analysis.

### Histology

Formalin-fixed tumors from tumor-bearing mice were paraffin-embedded and cut in 4-μm sections, de-paraffinized, and exposed to rabbit anti human TNF Receptor I antibody (Abcam Cat# ab19139, RRID : AB_2204128), mouse anti human TNF Receptor II antibody (Abcam Cat# ab8161, RRID : AB_306318), Dako EnVision+System-HRP Labelled Polymer anti-rabbit (Dako Cat# K4002) and anti-mouse (Dako Cat# K4000). Mayer’s hematoxylin was used for counterstaining. Negative controls, exposed to the labelled polymers only, showed no staining.

Images of 10 areas of each tumor section from 3 mice each treatment group were acquired on an Olympus BX43 microscope ×40/0.75 magnification. For collagen content Trichrome stain kit (Abcam, Cat# ab 150686) was used according to the manufacturer’s protocol.

### Protein Quantifications

The microdialysis samples were analyzed using a multiplex proximity extension assay (PEA, Olink Bioscience, Uppsala Sweden) as previously described (29–31). In brief, 1 μL sample was incubated with proximity antibody pairs tagged with DNA reporter molecules. The DNA tails formed an amplicon by proximity extension, which was quantified by high-throughput real-time PCR (BioMark™ HD System; Fluidigm Corporation, South San Francisco, CA, USA). The generated fluorescent signal correlate with protein abundance by quantitation cycles (Cq) produced by the BioMark Real-Time PCR Software. To minimize variation within and between runs, the data were normalized using both an internal control (extension control) and an interplate control and transformed using a predetermined correction factor. The pre-processed data were provided in the arbitrary unit normalized protein expression (NPX) on a log_2_ scale, which were then linearized by using the formula 2^NPX^. A high NPX value corresponded to a high protein concentration. Values represented a relative quantification meaning that no comparison of absolute levels between different proteins could be made.

### Estradiol Analysis

Estradiol levels in the microdialysis samples were analyzed using a high sensitivity immunoassay kit (DRG International, Springfield Township, NJ, USA).

### Statistical Analyses

Statistical analyses were performed using nonparametric Wilcoxon matched-pairs signed rank tests or Kruskal Wallis tests followed by unpaired Mann-Whitney U tests when more than two groups were compared as the data was non-normally distributed. Spearman’s correlation test was used for calculations of correlations. A *P*<0.05 was considered statistically significant. Statistics were performed with Prism 9.0 (GraphPad, San Diego, CA, USA).

## Results

### TNFSF/sTNFRSF Family of Proteins in Human Breast Cancer

**Figure 1:**
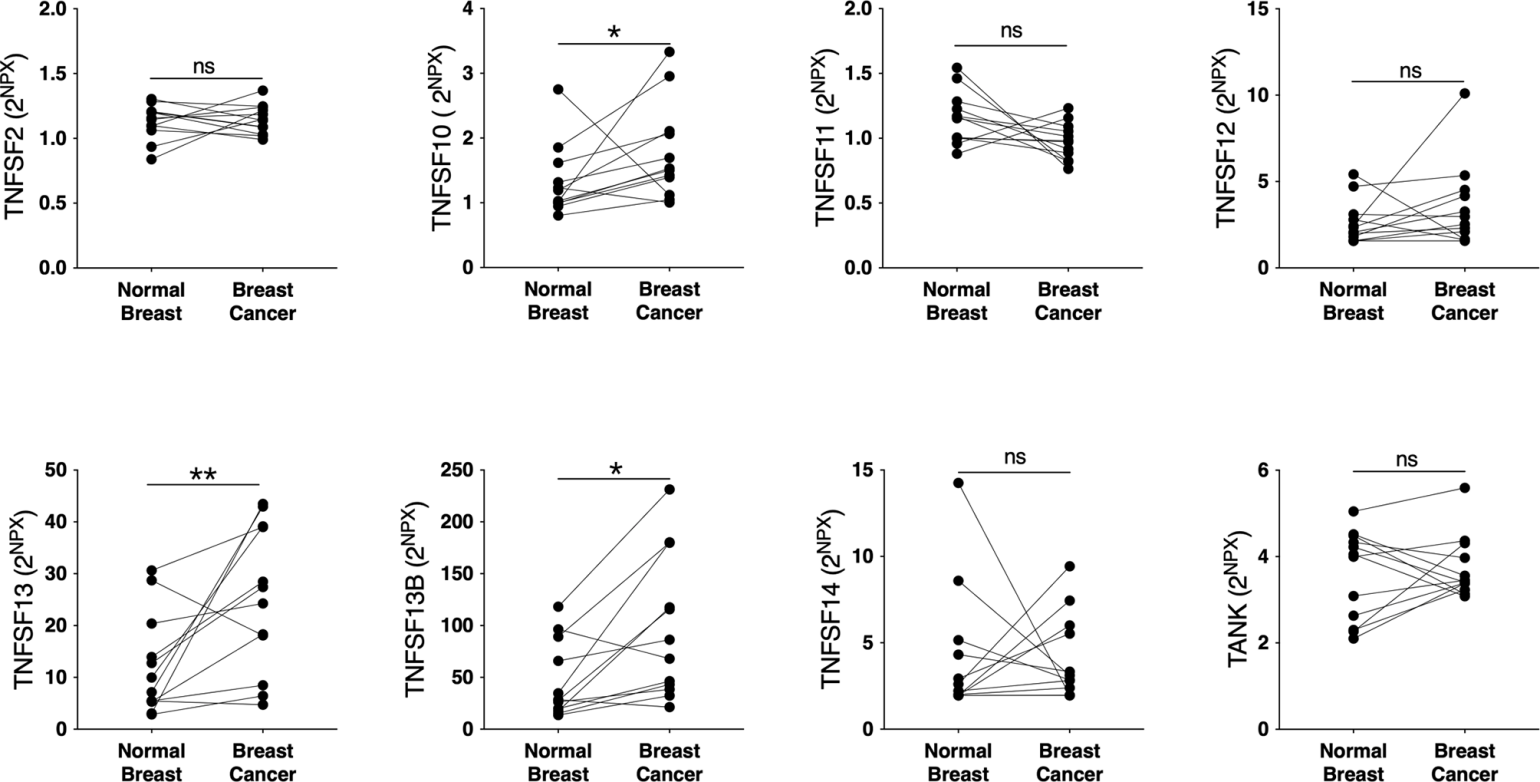
Extracellular levels of TNFSF in vivo in breast cancers and adjacent normal breast tissue. 12 patients with estrogen receptor positive breast cancer underwent microdialysis before surgery. One catheter was inserted into the breast cancer and another into adjacent normal breast tissue. Proteins were quantified using proximity extension assay. Data represents protein abundance in linear values (2^NPX^ as described in the materials and methods section). *P < 0.05, **P < 0.01, ns, not significant.

**Figure 2:**
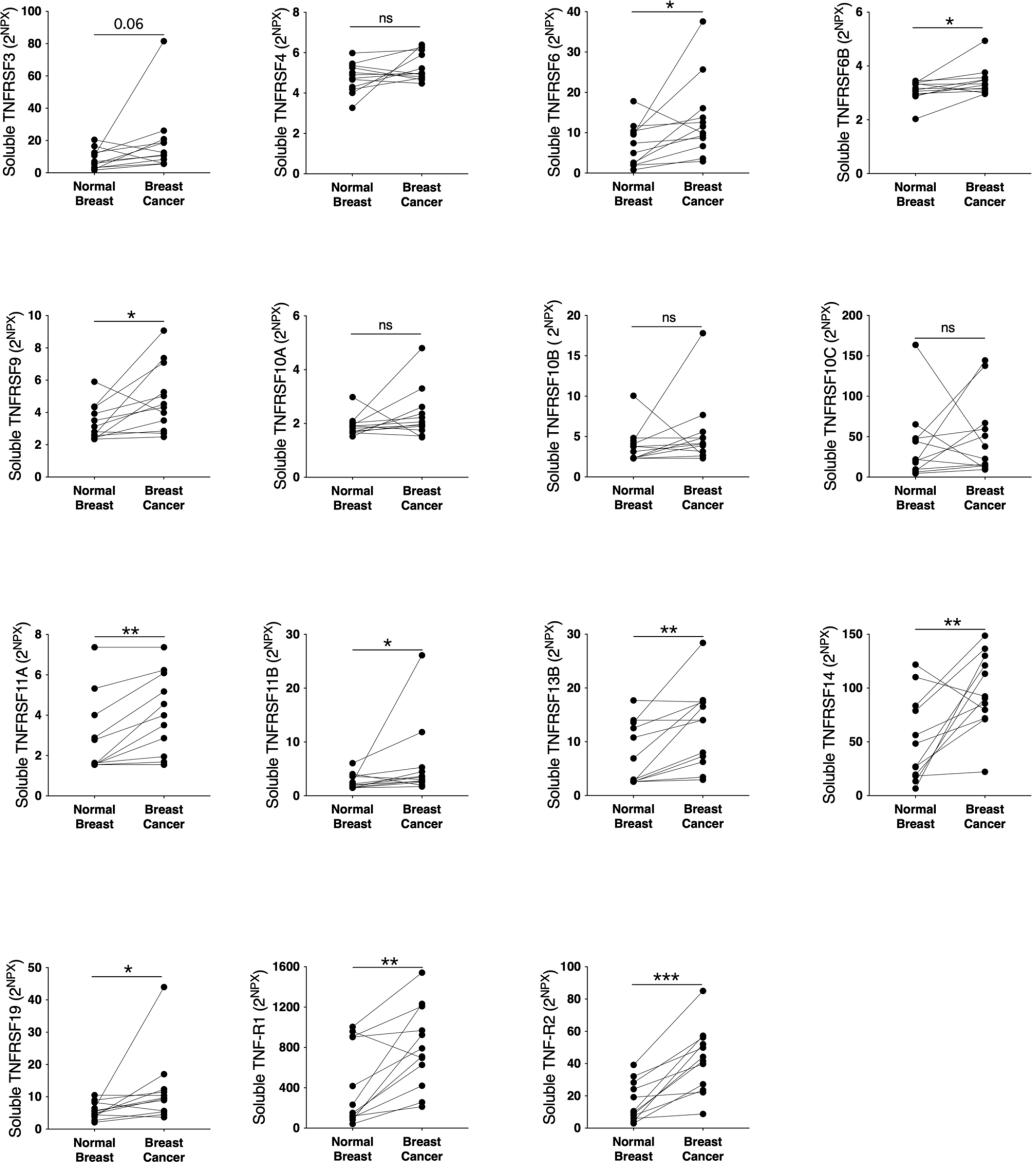
Extracellular levels of soluble TNFRSF in vivo in breast cancers and adjacent normal breast tissue. 12 patients with estrogen receptor positive breast cancer underwent microdialysis before surgery. One catheter was inserted into the breast cancer and another into adjacent normal breast tissue. Proteins were quantified using proximity extension assay. Data represents protein abundance in linear values (2^NPX^ as described in the materials and methods section). *P < 0.05, **P < 0.01, ***P < 0.001, ns, not significant.

To elucidate whether the TNFSF/sTNFRSF family of proteins were affected, and therefore possible drug targets, in human breast cancer we performed microdialysis in women with breast cancer prior to their surgery to retrieve extracellular molecules in live tissues. The women with breast cancer were included regardless of their breast density, which was undetermined. As shown in **Figure 1**, TNFSF10, -13 and -13B were upregulated whereas TNFSF2, -12, -14, and TANK were unaffected in breast cancer compared to normal adjacent breast tissue. Regarding the soluble receptors the levels of sTNFRSF6, -6B, -9, -11A, -11B, -13B, -14, -19 and sTNF-R1 and -R2 were significantly increased in breast cancers whereas sTNFRSF4, -10A, -10B and -10C were unaffected, **Figure 2**. Borderline significance of sTNFRSF3 (p=0.06) were demonstrated, **Figure 2**.

### TNFSF/sTNFRSF Family of Proteins in Normal Human Breast Tissues

**Figure 3:**
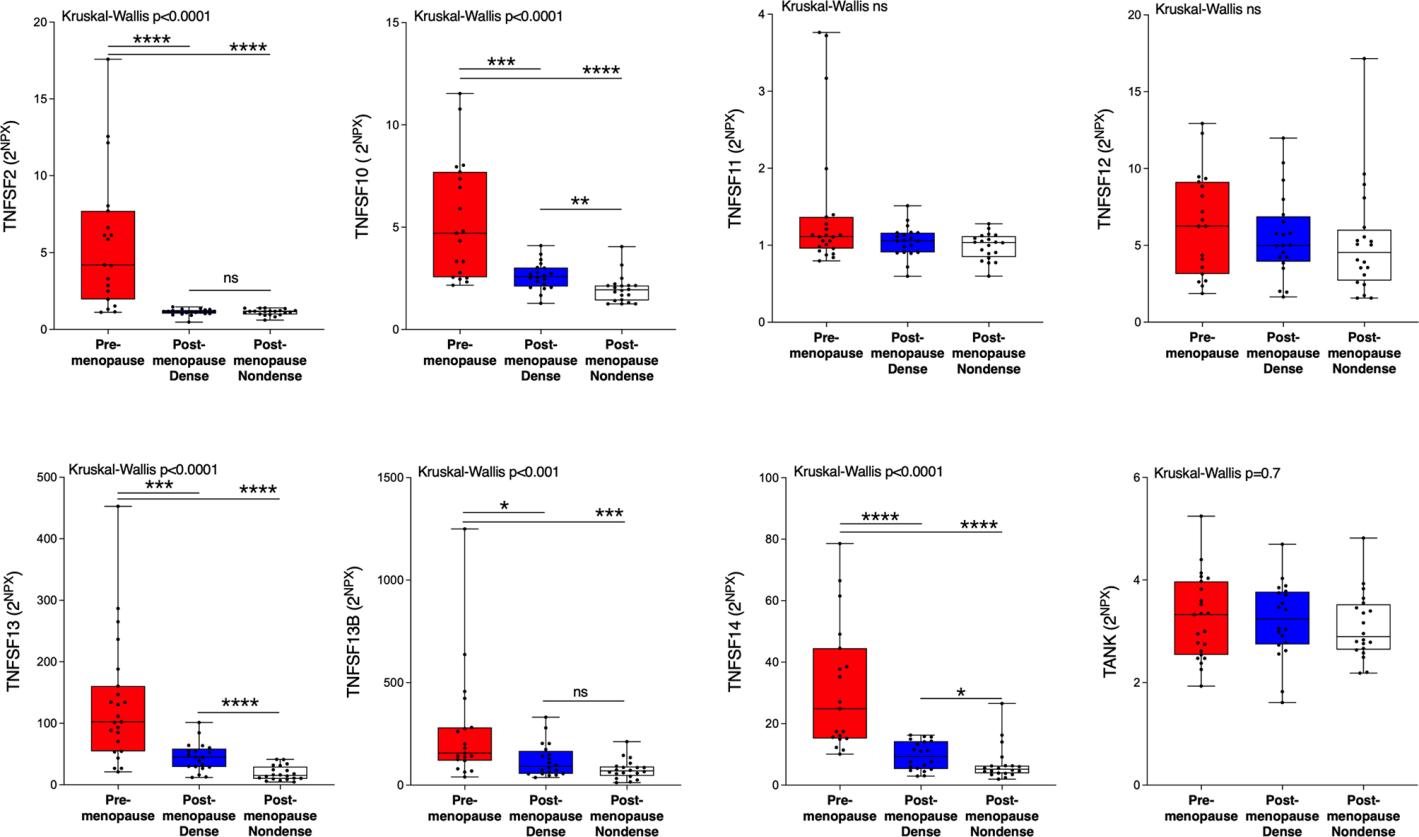
Extracellular levels of TNFSF *in vivo* in healthy normal breast tissue of premenopausal women and postmenopausal women with different breast densities. A total of 59 women underwent microdialysis of their left breast; 40 postmenopausal healthy volunteer women, attending the regular mammography-screening program categorized as either having dense (n = 20, blue boxes) or nondense breasts (n = 20, clear boxes) and 19 premenopausal women (red boxes). Proteins were quantified using proximity extension assay. Data represents extracellular local protein abundance in linear values (2^NPX^ as described in the Methods section). A significant Kruskal-Wallis was followed by Mann Whitney U-test. Data are displayed as box plots with median and 10–90 percentile. *P < 0.05, **P < 0.01, ***P < 0.001, ****P < 0.0001, ns=not significant.

**Figure 4:**
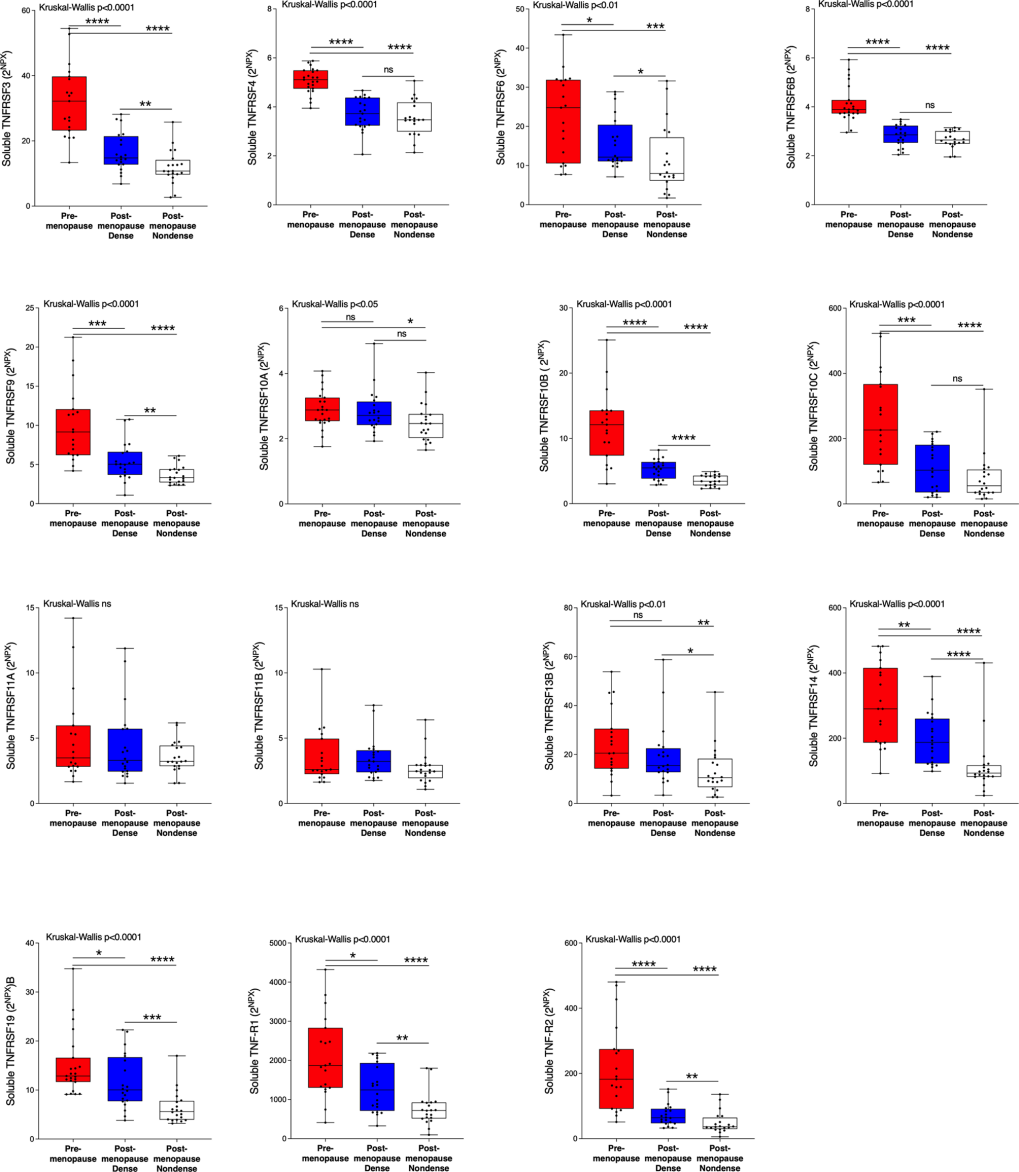
Extracellular levels of soluble TNFRSF *in vivo* in healthy normal breast tissue of premenopausal women and postmenopausal women with different breast densities. A total of 59 women underwent microdialysis of their left breast; 40 postmenopausal healthy volunteer women, attending the regular mammography-screening program categorized as either having dense (n = 20, blue boxes) or nondense breasts (n = 20, clear boxes) and 19 premenopausal women (red boxes). Proteins were quantified using proximity extension assay. Data represents extracellular local protein abundance in linear values (2^NPX^ as described in the Methods section). A significant Kruskal-Wallis was followed by Mann Whitney U-test. Data are displayed as box plots with median and 10–90 percentile. *P < 0.05, **P < 0.01, ***P < 0.001, ****P < 0.0001, ns, not significant.

Next, we wanted to investigate whether the TNFSF/sTNFRSF proteins were affected in normal breast tissue with inherently high risk of breast cancer, dense breast tissue of postmenopausal women, as compared to nondense breasts with inherently low risk of breast cancer. Additionally, to be able to investigate whether estradiol affects these proteins a group of premenopausal women were included. As shown in **Figure 3**, 5 ligands, TNSFS2, -10, -13, -13B and 14, were significantly increased in premenopausal breasts as compared to both dense and nondense breasts in postmenopausal women. Furthermore, TNFSF10, -13 and -14 were increased in dense breasts compared to nondense breasts in postmenopausal women, **Figure 3**. No significant changes were detected of TNFSF11, -12 and TANK, **Figure 3**. Regarding the soluble receptors, 11 were increased in premenopausal breast tissue compared to both dense and nondense postmenopausal breasts; sTNFRSF3, -4, -6, -6B, -9, -10B, -10C, -14, -19, and TNF-R1 and R2, **Figure 4**. Furthermore, sTNFRSF3, -6, -9, -10B, -13B, -14, -19, and TNF-R1 and R2 were increased in dense breasts compared to nondense breasts, **Figure 4**. No significant changes were detected of sTNFRSF11A and 11B, **Figure 4**.

### Correlations Between TNFSF/sTNFRSF Proteins With Estradiol and LTF Locally in Breast Tissue

**Figure 5:**
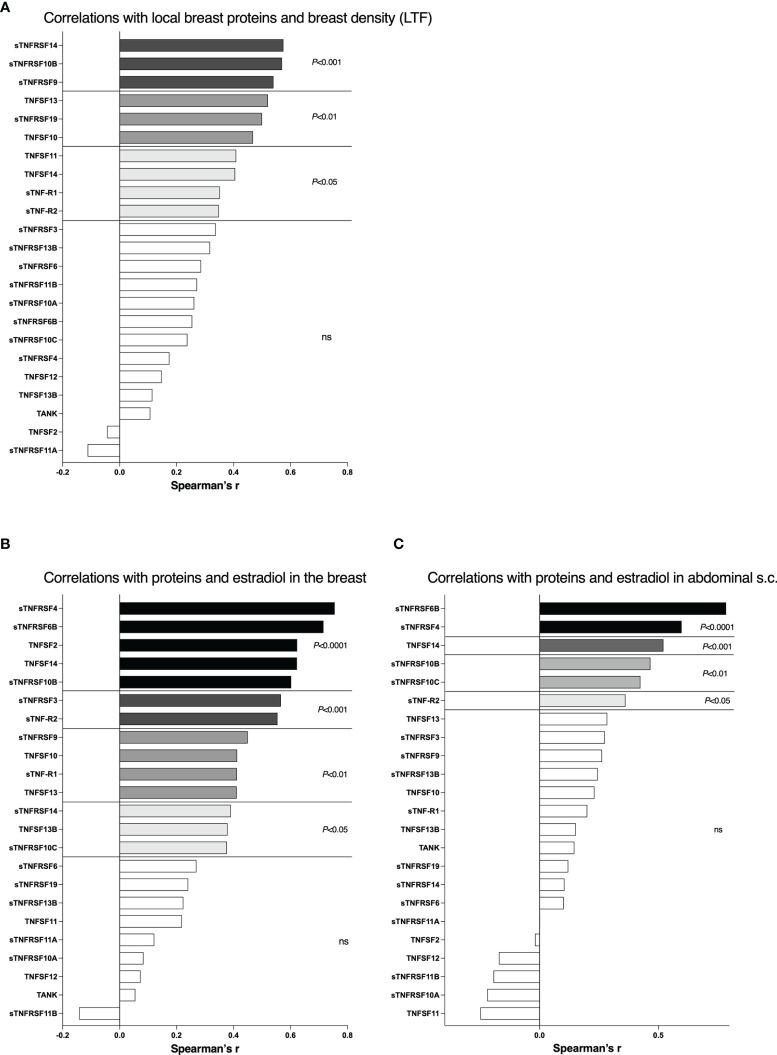
Correlations between local breast levels of soluble TNFRSF and TNFSF with breast density and local breast estradiol levels. A total 40 postmenopausal healthy volunteer women, attending the regular mammography-screening program and 19 healthy premenopausal women underwent microdialysis of their left breast for sampling of extracellular proteins *in vivo*. Proteins and estradiol (E2) were quantified in the microdialysate as described in the materials and methods section. Breast density was quantified as lean tissue fraction (LTF) determined in the MRIs. **(A)** Correlations of local breast levels of TNFSFs/sTNFRSFs and LTF in postmenopausal women. **(B)** Correlations of local breast levels of TNFSFs/sTNFRSFs and local breast estradiol levels. Premenopausal women and postmenopausal women with dense breasts were included. **(C)** Correlations of local levels of TNFSFs/sTNFRSFs and local estradiol levels in abdominal s.c. fat. Bars represent Spearman’s Rank correlation coefficient. White bars, not significant.

There were no differences in local breast estradiol levels in the postmenopausal women with nondense or dense breast tissue, 36 (16-69) pmol/l in nondense vs. 38 (14-74) pmol/l in dense breasts (median (range)). As the estradiol levels were similar in both postmenopausal groups correlations with breast density measured by LTF and proteins in TNFSF/sTNFRSF family was possible to perform. As shown in **Figure 5A**, 10 of the 23 proteins correlated significantly with LTF whereof sTNRSF14, -10B, and -9 being the most significant, p<0.001.

The premenopausal women, which per definition have dense breasts, exhibited significantly increased local breast estradiol levels, 205 (73-264) pmol/l, compared to postmenopausal women with dense breasts. Thus, in these two groups correlations depending on estradiol were possible to perform as both groups represent dense breast tissue. 14 of the 23 proteins correlated significantly with estradiol whereof sTNFRSF4, -6B, TNF, TNFSF14 and sTNFRSF10B being highly significant, p<0.0001, **Figure 5B**. As a measure of systemic effects, micodialysis was used to collect extracellular factors from abdominal s.c. fat in all healthy volunteers. By this approach breast tissue specific alterations can be deciphered. As shown in **Figure 5C**, 6 of the proteins correlated significantly with local fat estradiol whereof sTNFRSF6B and sTNFRSF4 being highly significant, p<0.0001.

### TNFSF/sTNFRSF Proteins in Abdominal s.c. Fat

**Figure 6:**
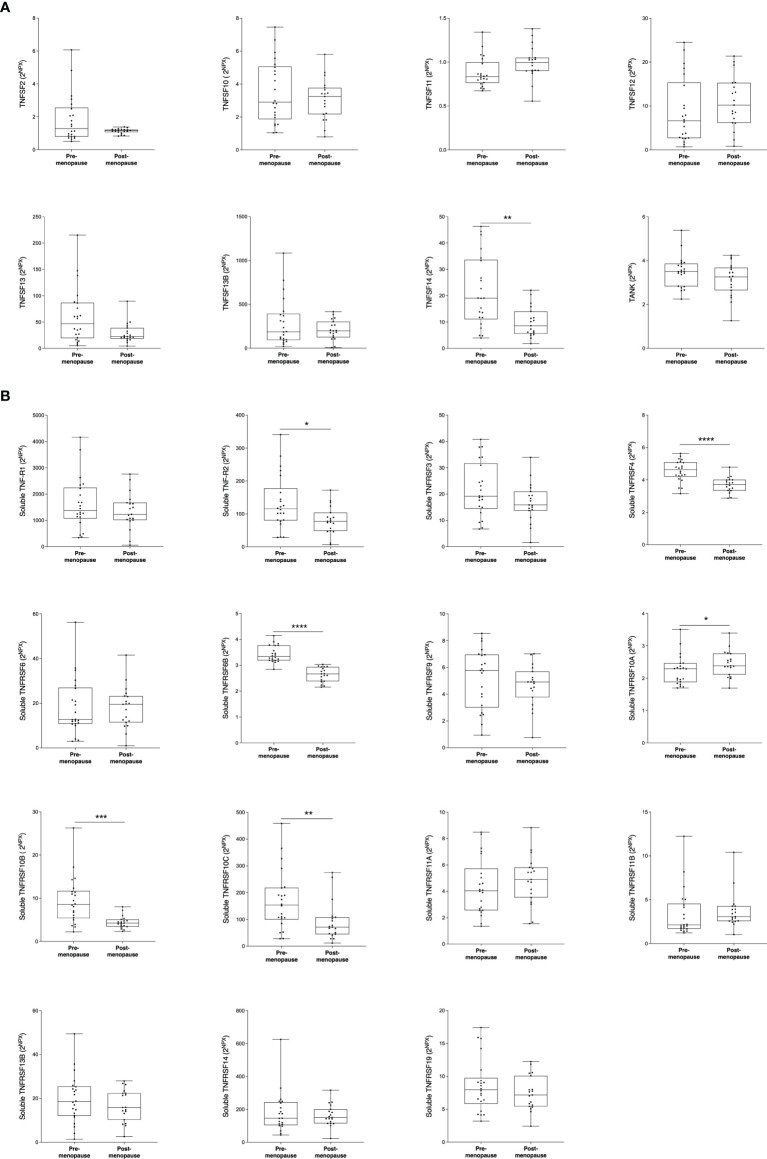
Local levels of TNFSF/sTNFRSF in abdominal s.c. fat. The women described in **Figure 5** were subjected to microdialysis of the abdominal s.c. fat at the same time point as the microdialysis of breast tissue. Proteins were quantified in the microdialysate as described in the materials and methods section. Mann Whitney U-test was used. Data are displayed as box plots with median and 10–90 percentile. *P < 0.05, **P < 0.01, ***P < 0.001, ****P < 0.0001. **(A)** TNF ligands (TNFSFs) **(B)** Soluble TNF receptors (sTNFRSFs).

The specific data from s.c. abdominal fat were as follows; local estradiol levels were 36 (27-60) pmol/l in postmenopausal women vs. 202 (88-270) pmol/l in premenopausal women (median (range)), p<0.0001. As shown in **Figure 6A** among the ligands only TNFSF14 was significantly increased in premenopausal women. Among the soluble receptor shown in **Figure 6B**, sTNF-R2, sTNFRSF4, -6B, 10A, 10B, and 10C were significantly increased in premenopausal women.

### TNFSF2-TNFR in Experimental Breast Cancer

**Figure 7:**
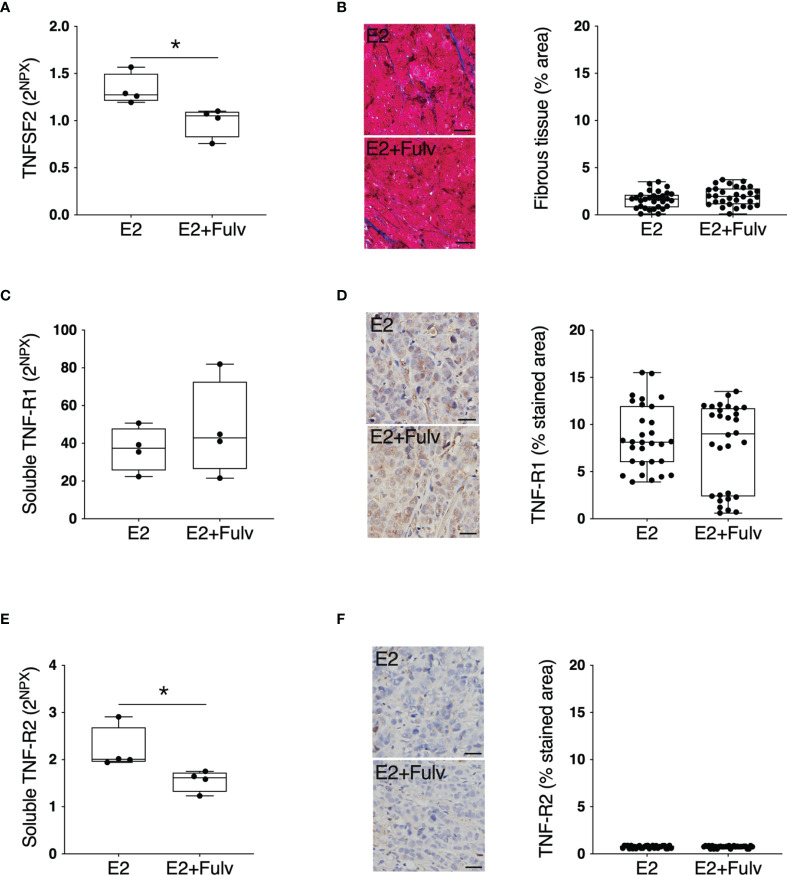
TNFSF2, TNF-R1, and TNF-R2 in experimental ER+ breast cancer. Oophorectomized athymic mice supplemented with physiological levels of estradiol (E2) were injected with MCF-7 into the dorsal mammary fat pads. At similar tumor sizes, mice either continued with E2 or were additionally treated with fulvestrant (E2+Fulv) (5 mg/mouse every 3 days, s.c.). Size-matched tumors from the different treatment groups underwent microdialysis for sampling of extracellular proteins *in vivo*, which were quantified using proximity extension assay. Data represents extracellular local protein abundance in linear values (2^NPX^ as described in the Methods section). Tumor sections were subjected to immunohistochemistry. Data are presented as the mean ± SD. *P < 0.05. **(A)** Extracellular TNFSF2, n = 4 animals per group. **(B)** Tumor sections from each treatment group were stained for collagen (blue) and quantified as the percentage of area with positive staining. Representative sections are depicted. Scale bar = 20 µm. **(C)** Soluble TNF-R1, n = 4 animals per group. **(D)** Tumor sections from each treatment group were stained for TNF-R1 and quantified as the percentage of area with positive staining. Representative sections are depicted. Scale bar = 20 µm. **(E)** Soluble TNF-R2, n = 4 animals per group. **(F)** Tumor sections from each treatment group were stained for TNF-R2 and quantified as the percentage of area with positive staining. Representative sections are depicted. Scale bar = 20 µm.

Next, we set up experimental estrogen receptor positive (ER+) breast cancer in mice to investigate possible estrogen regulation of extracellular TNFSF2 and its main receptors TNF-R1 and TNF-R2 in cancerous tissue. As shown in **Figure 7A** treatment with the pure anti-estrogen fulvestrant significantly decreased the *in situ* levels of extracellular TNFSF2. The tumors comprised very little stroma with no differences between the groups, **Figure 7B**. No differences were found in soluble or cellular TNF-R1, **Figures 7C, D**. Additionally, soluble TNF-R2 was significantly decreased by fulvestrant therapy whereas no significant difference was found of cellular TNF-R2, **Figures 7E, F**.

## Discussion

To the best of our knowledge no previous data on soluble members of the TNFSF/TNFRSF family of proteins in breast cancer and normal breast tissue with high risk of breast cancer has been studied. As summarized in **Table 1**, we demonstrate that most of these proteins were significantly increased in human breast cancers compared to normal adjacent breast tissue. In postmenopausal women dense breasts, as compared to nondense breasts, exhibited similar alterations as breast cancers. Additionally, breast tissue in premenopausal women exhibited significant different levels of many of the TNFSF/TNFRSF proteins compared to both breast tissue types of postmenopausal women. Our data revealed that breast density correlated significantly with 10 of the 23 proteins whereas estradiol exhibited significant positive correlations with 14. An estrogen dependent regulation of soluble extracellular TNF and TNF-R2 was corroborated in experimental ER+ breast cancer in mice.

**Table 1:** Microdialysis was performed in breast tissue in women and proteins and estradiol were quantified in the dialysates.

Protein	Breast cancer vs. normal breast	Dense vs. nondense breast tissue	Correlation with local breast estradiol	Correlation with local breast LTF
TNFSF2			**Yes**	
TNFSF10	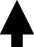	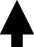	**Yes**	**Yes**
TNFSF11				**Yes**
TNFSF12				
TNFSF13	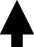	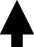	**Yes**	**Yes**
TNFSF13B	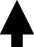		**Yes**	
TNFSF14		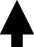	**Yes**	**Yes**
TANK				
sTNFRSF3		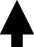	**Yes**	
sTNFRSF4			**Yes**	
sTNFRSF6	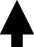	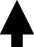		
sTNFRSF6B	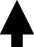		**Yes**	
sTNFRSF9	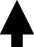	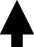	**Yes**	**Yes**
sTNFRSF10A				
sTNFRSF10B		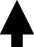	**Yes**	**Yes**
sTNFRSF10C			**Yes**	
sTNFRSF11A	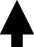			
sTNFRSF11B	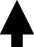			
sTNFRSF13B	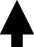	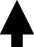		
sTNFRSF14	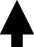	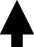	**Yes**	**Yes**
sTNFRSF19	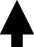	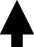		**Yes**
sTNFR-1	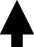	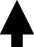	**Yes**	**Yes**
sTNF-R2	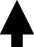	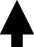	**Yes**	**Yes**

Magnetic resonance imaging was used for quantifications of lean tissue fraction (LTF), a continuous measure of breast density.

TNFSF/TNFRSF families comprise 19 ligands that can bind to one or more of 29 structurally similar receptor proteins (32, 33). These proteins play an important role in various biological processes and aberrant production and receptor signaling by TNFSF/TNFRSF has been associated with the pathogenesis of several inflammatory diseases and cancer (11). Most members of the TNFRSF contain transmembrane (TM) domains and by proteolytic processing of these, soluble ligand-binding molecules from the receptors are formed, sTNFRSF. Local accumulation of the soluble forms of receptors and ligands will interfere with the binding of membrane-bound forms and act both as inhibitors and buffering agents, which may lead to decreased intensity of signaling activation or an extended duration of signaling. TNF signaling can also be modulated by binding of TNFs to decoy receptors such TNFRSF6B, TNFRSF10C, TNFRSF10D, and TNFRSF11B (11).

Due to the initial observations that TNF could cause tumor necrosis TNF therapy was tested in clinical trials of cancer. Due to systemic toxicity these were, however, terminated and TNF is now only approved in a loco-regional setting as a cancer therapeutic (34, 35). Later studies have revealed that the TNF signaling is complex and somewhat contradictory. In preclinical models of breast cancer, TNF signaling may promote migration and invasion of breast cancer cells, as well as to induce apoptosis and exert cytotoxic effects *in vitro* (34). For example, when TNFSF2 binds to TNF-R1 and TNF-R2 apoptosis may be stimulated but both receptors have also been shown to activate proliferation by the NF-kB pathway (34). Additionally, TNFSF6 may be proapoptotic when binding to TNFRSF6 by activating caspase 8 complex whereas apoptosis is inhibited by its binding to TNFRSF6B (36). TNFRSF6B itself can attenuate inflammation and overexpression of this proteins has been related to breast cancer progression (37, 38). TNFSF10 is another ligand with both pro- and anti-apoptotic effects as well as effects on the immune response (39). These data have been corroborated by genome wide screens that recently uncovered the TNF-pathway as essential for susceptibility of immunotherapy and important for the understanding of the immune response in cancer (40). Specifically, TNFSF13 and TNFSF14 can increase the effects of immunotherapy by facilitating immune cell mobilization into the cancer (41–43). As recently reviewed, several members of TNFSF/TNFRSF families are key for the coordination of mechanism that result in activation or inhibition of the immune response in both inflammatory diseases as well as in cancer (33). Most members of the TNFRSF family are involved in apoptosis regulation and activation of inflammation *via* NF-κB activation; TNFRSF3 and 6 may induce apoptosis as well as activate the immune response by increasing secretion of inflammatory cytokines including IL-8 as well as induce apoptosis of tumor cells (44, 45). TNFRSF9 increase inflammation, affect the T-cell population as well as increase proliferation of peripheral monocytes (46). Similar to other members of the TNFRSF family, TNFRSF10B and 10C can induce inflammation and may either enhance or inhibit apoptosis depending on the expression of other cytokines in the microenvironment (47). TNFRSF14 is expressed on a broad range of cancer cells and may activate the inflammatory response, which has implicated its role in autoimmune pathogenesis (48). TNFRSF19 may be involved in beta-catenin regulation of NF-κB and has primarily been associated with melanoma, glioblastoma, and colorectal cancer (49, 50). However, further studies of the specific roles of the different TNFSFs/TNFRSFs in mammographic density or breast cancer progression need to be performed.

The role of estrogen in the regulation of TNFSF2 is somewhat controversial and there is no consensus in the literature. In bone tissue, estradiol has been shown to downregulate TNFSF2 (51). Contrary to this, it has been demonstrated that estradiol up-regulates TNFSF2 in dendritic cells in mice and in synovial tissue of humans (52, 53). Additionally, anti-estrogen decreases the levels of TNFSF2 in experimental models of breast cancer (54). Our data revealed a significant positive correlation between estradiol and TNFSF2 determined locally in normal breast tissue supporting an estrogen dependent regulation in the mammary gland. The data from normal breast tissue was corroborated by our results from experimental ER+ breast cancer where treatment with the pure anti-estrogen fulvestrant significantly decreased the levels of extracellular *in vivo* TNF. Surprisingly, in ER+ human breast cancers no increased levels, as compared to normal adjacent breast tissues, of TNFSF2 were detected. However, all samples from the breast cancer patients were in the lower range of the detection limit of the assay for TNFSF2 and may therefore not accurately reflect possible differences. Increased sensitivity of the assay may yield different results and this needs to be performed before any conclusions can be made regarding extracellular levels of TNFSF2 in human breast cancer. How or if estradiol may affect other members of the TNFSF/TNFRSF family is less studied. Estradiol has been shown to increase the expression of TNFSF2, TNF-R1, and TNF-R2 in pituitary cells and fibroblasts from breast tissue (55, 56). Our present data are in line with these previous findings as TNF and soluble TNF-R1 and soluble TNF-R2 correlated significantly with estradiol in normal breast tissue and exhibited increased levels in premenopausal breast tissue as compared to postmenopausal breasts. Regarding TNFSF14, previous data suggest that ovariectomy induces an increase of the protein in bone and circulating T-cells in women (57),. These data contrast with our findings where there was a highly significant positive correlation between local estradiol and TNFSF14 and over twice as high levels in premenopausal breasts compared to postmenopausal breast tissue. In bone tissue estradiol has been found to regulated TNFRSF11B at the transcriptional levels (58). However, our data do not support an estrogen regulation of TNFRSF11B in breast tissue as no differences were found between pre- and postmenopausal women an no correlations with estradiol was detected.

To elucidate whether our findings of correlations between estradiol and several of the TNFSFs/TNFRSFs were breast tissue specific we also measured these proteins in abdominal s.c. fat. Our data suggest that estradiol may affect levels of sTNFRSF6B, sTNFRSF4, TNFSF14, sTNFRSF10B, sTNFRSF10C, and sTNF-R2 systemically as these proteins correlated with estradiol both in breast tissue and abdominal s.c. fat. The levels of TNFSF2, sTNFRSF3, sTNFRSF9, TNFSF10, sTNF-R1, TNFSF13, sTNFRSF14, TNFSF13B, however, correlated with estradiol in breast tissue only suggesting that these factors may be under hormonal control in a tissue specific manner.

The relationship between mammographic density and the TNFSF/TNFRSF family of proteins is to a large extent unexplored and limited data are available. Immunohistochemical determination of TNFSF2 has failed to show any association with mammographic density (59), which is in line with our data not showing any association with breast density quantified by LTF and TNFSF2.

TNFSF/TNFRSF family of proteins may induce both pro-tumorigenic and tumor-suppressive effects in various tissues and organs. Further studies are warranted to fully understand these pathways in breast cancer and normal breast tissue at high risk of cancer. Our data clearly show that these proteins are dysregulated in both breast cancer and dense breast tissue of postmenopausal women and that several of the proteins are under estrogen control in breast tissue. Our data indeed suggest these pathways may be worthwhile to further elucidate as targets for therapy and prevention of breast cancer.

## Data availability Statement

The original contributions presented in the study are included in the article/supplementary material. Further inquiries can be directed to the corresponding author.

## Ethics Statement

The studies involving human participants were reviewed and approved by Regional Ethical Review Board of Linköping. The patients/participants provided their written informed consent to participate in this study. The animal study was reviewed and approved by The Institutional Animal Ethics Committee at Linköping University.

## Author contributions

CD designed the project and performed all microdialysis investigations. AA carried out sample preparation and participated in the animal work. CD, JE, MZ, AA, PL, MF analyzed the data and prepared and finally approved the manuscript.

## Funding

This work was supported by grants to C.D. from the Swedish Cancer Society (2018/464), the Swedish Research Council (2018-02584), LiU-Cancer, and ALF of Linköping University Hospital.

## Conflict of Interest

The authors declare that the research was conducted in the absence of any commercial or financial relationships that could be construed as a potential conflict of interest.

## Publisher’s Note

All claims expressed in this article are solely those of the authors and do not necessarily represent those of their affiliated organizations, or those of the publisher, the editors and the reviewers. Any product that may be evaluated in this article, or claim that may be made by its manufacturer, is not guaranteed or endorsed by the publisher.
